# Strategies for Annulus Fibrosus Regeneration: From Biological Therapies to Tissue Engineering

**DOI:** 10.3389/fbioe.2018.00090

**Published:** 2018-07-10

**Authors:** Genglei Chu, Chen Shi, Huan Wang, Weidong Zhang, Huilin Yang, Bin Li

**Affiliations:** ^1^Orthopaedic Institute, Medical College, Soochow University, Suzhou, Jiangsu, China; ^2^Department of Orthopaedic Surgery, First Affiliated Hospital, Soochow University, Suzhou, China; ^3^Department of Biomedical Engineering, National University of Singapore, Singapore, Singapore; ^4^China Orthopaedic Regenerative Medicine Group, Hangzhou, China

**Keywords:** intervertebral disc, annulus fibrosus, tissue regeneration, biomolecular therapy, gene therapy, mechano-regulation, scaffolds, tissue engineering

## Abstract

Intervertebral disc (IVD) is an avascular tissue which contributes to the weight bearing, motion, and flexibility of spine. However, IVD is susceptible to damage and even failure due to injury, pathology, and aging. Annulus fibrosus (AF), the structural and functional integrity of which is critically essential to confine nucleus pulpous (NP) and maintain physiological intradiscal pressure under mechanical loading, plays a critical role in the biomechanical properties of IVD. AF degeneration commonly results in substantial deterioration of IVD. During this process, the biomechanical properties of AF and the balance between anabolism and catabolism in IVD are progressively disrupted, leading to chronic back pain, and even disability of individuals. Therefore, repairing and regenerating AF are effective treatments to degeneration-associated pains. However, they remain highly challenging due to the complexity of natural AF tissue in the aspects of cell phenotype, biochemical composition, microstructure, and mechanical properties. Tissue engineering (TE), by combining biological science and materials engineering, shed lights on AF regeneration. In this article, we review recent advances in the pro-anabolic approaches in the form of cell delivery, bioactive factors delivery, gene therapy, and TE strategies for achieving AF regeneration.

## Introduction

Low back pain (LBP) is one of the most common causes of activity limitations, neurological deficit, and disability in affected individuals. In the United States, more than 52 million people suffer from back pain and the clinical cost reaches more than $40 billion each year (Murray et al., 2012). Intervertebral disc (IVD) disorders contribute to LBP and neck pain in multiple ways with few available treatments. Current conservative treatments to LBP include nonpharmacological and pharmacological approaches, and surgical approaches may be considered if conservative methods fail (Finch, 2006). However, surgical interventions such as spinal fusion are very invasive and often require a long postoperative recovery, with a non-negligible risk of complications high post-surgery recurrence rate (Andersson, 1999). Therefore, new approaches are urgently needed for the treatment of degeneration disc disease (DDD).

**Figure 1:**
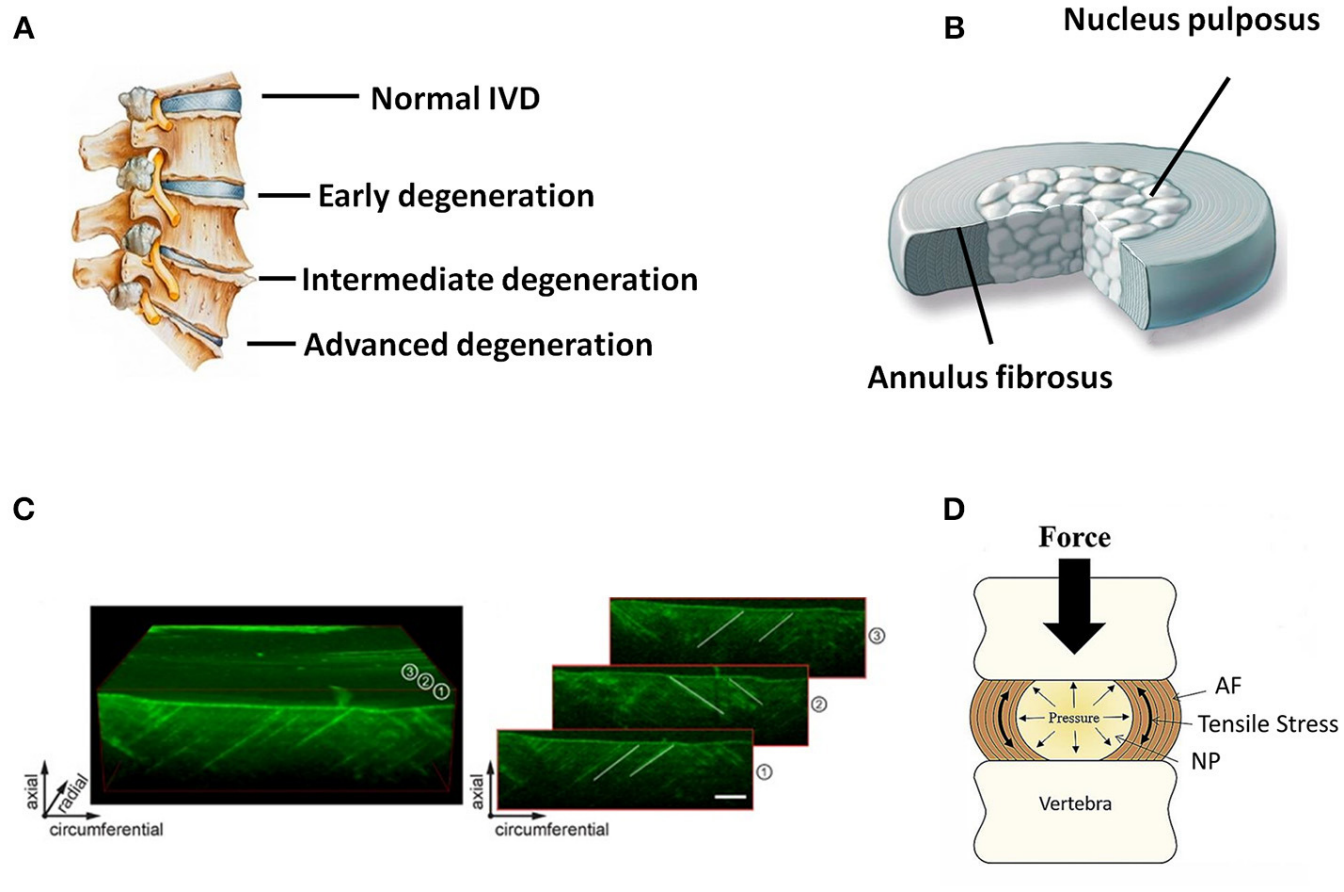
**(A)** Schematic illustration of normal and degenerative IVDs. **(B)** Schematic illustration of the anatomic structure of an IVD. **(C)** 3D representation of an ovine AF using optical coherence tomography. Individual images on the right side show how collagen orientation changes along radial direction. Scale bar, 500 μm (Reprinted with permission from Han et al., 2015). **(D)** Forces and motion contribute to axial loading of IVD that is balanced by the hydrostatic pressure generated in the gelatinous NP. Pressure in NP causes tensile stress in the surrounding AF or “hoop stress” (Reprinted with permission from Bowles and Setton, 2017).

Anatomically, the IVD is the fibrocartilaginous part of a three-component construct that consists of two posterior articulations of diarthrodial facet joints, along with a gelatinous proteoglycan-rich nucleus pulposus (NP) in the center and a multi-lamellar collagen-rich fibrocartilage annulus fibrosus (AF) in the surrounding, both of which are connected by the cartilaginous endplates (CE) to the vertebral bodies (Figure 1A). Together, these constructs contribute to the weight bearing, motion, and flexibility of spine while protecting its neural anatomy. Changes in the biomechanical and structural properties of IVD negatively impact motion, spinal alignment, flexibility, or neural anatomy, which can ultimately result in loss of normal function of IVD and further serious degenerative diseases like DDD. In the past decades, diverse strategies have been developed aiming to ameliorate IVD degeneration and promote its regeneration (Buckwalter, 1995; Adams and Dolan, 2012). While considerable progress has been achieved in treatment and regeneration of NP, much less is achieved in that of AF. As a crucial supporting component in the biomechanical constitution of IVD, the structural and mechanical integrity of AF is highly essential in confining NP, and tears or fissures in AF are closely associated with the onset and development of DDD (Nerurkar et al., 2009; Li et al., 2017). Therefore, strategies to repair or regenerate of AF are now considered necessary in DDD treatments.

Recent progress in understanding of AF physiopathology from aspects of cell biology, biomaterials, and mechanics, is leading to new approaches towards AF repair and regeneration (Driscoll et al., 2013). Unlike NP, AF is a typical inhomogeneous tissue, with its cellular and biochemical characteristics varying peripherally from inner to outer zones along its radial direction, which, in turn, lead to region-specific biological and biomechanical functions (Bruehlmann et al., 2002; Cortes et al., 2013). Therefore, successful AF repair and regeneration require the recovery of biomechanical and structural properties of healthy AF and restoration of biological behaviors of resident cells within AF. In view of the cells apoptosis during AF degeneration, cell-based therapies were studied with growing interest. Recently, gene mutations or microRNA dysregulation was also regarded as a therapeutic approach for AF-associated diseases based on the delivery of genes or small interfering RNA (siRNA) to stimulate the restoration of AF functions (Clouet et al., 2018). Moreover, owing to the tremendous complexity of AF tissue, the use of biological factors targeting AF degeneration was also contemplated. Tissue engineering (TE) approaches are now emerging as promising therapy toward DDD using engineered disc and AF replacements. An ideal engineered AF tissue should recapitulate the biochemical, microstructural, and cellular complexity of native AF tissue (Chan et al., 2011). Although few reviews have been published on delivery systems for the treatment of AF defects, no systematic studies have described support structures with mechanical properties and structural features matching those of the AF (Sharifi et al., 2015; Li et al., 2016). In this review, we will first introduce the recent understanding in the pathophysiological mechanisms of AF and then focus on describing biomolecule-, gene-, cell-, and TE-based strategies for regeneration of AF.

## Structure and functions of AF

AF holds the NP by confining its swelling pressure and resisting shear and tensile stresses from the internal pressure exerted by the NP. In contrast to the relatively simple structure of NP, AF is a highly heterogeneous tissue in terms of cellular phenotype, biomechanics, biochemistry, and microstructure, with typical gradient characteristics along the radial direction. Microscopically, AF consists of 15–25 concentric layers, which are composed of alternatingly aligned oblique collagen fibers interspersed with proteoglycans (Figures 1B,C) (Bhattacharjee et al., 2012; Li et al., 2014). The lamellas in AF intercross with each other and possess prominent mechanical nonlinearity and anisotropy, contributing to the radially gradient mechanical properties of AF. The outer layers of AF are more oriented between layers than the inner ones. Moreover, the outer zone of AF is more fibrous consisting of mainly collagen-I, while the inner zone is more cartilaginous, containing mostly collagen-II, and aggrecan (Mizuno et al., 2004; Martin et al., 2014). Accordingly, the content of collagen-I increases from the inner zone to the outer zone of AF, and the opposite trend is found in the content of collagen-II and aggrecan. The collagen fibers of AF are tensioned by intradiscal pressure through two mechanisms: direct radial pressure from NP, and cranial-caudal stretch from the separation of the two endplates (Figure 1D). Besides the microstructural heterogeneity, the mechanical properties of AF also gradually change from inner to outer layers along radial direction, leading to regional specific mechanical properties and biological functions (Bowles et al., 2011; Wismer et al., 2014). In general, the elastic modulus of AF lamellae changes from 59 MPa to 136 MPa from the inner region to outer region of AF. This unique composition and architecture of AF is crucial for IVD to sustain anisotropic, nonlinear, and viscoelastic mechanical loading and maintain homeostasis. Therefore, the repair and regeneration of AF must take into consideration the structural and mechanical characteristics of this tissue over a wide range of length scales.

## Physiopathology of AF degeneration

AF degeneration involves complex and multifactorial physiopathology. Genetics, mechanical factors, environmental changes, aging, and toxic factors are considered as most relevant risk factors (Collin et al., 2017). Recent studies have highlighted that the changes in biomechanics and inflammation may play pivotal roles during AF degeneration (Adams et al., 2000; Urban et al., 2004).

Changes in biomechanics can lead to significant alteration of cellular physiology. Several groups showed that distinct compression on a spinal motion segment, either *in vivo* or *ex vivo*, could cause catabolic, anabolic and inflammatory cell responses in AF and IVD (Wang et al., 2007; Adams et al., 2009). As a result, cell behaviors in response to mechanical environmental variation are considered a pivotal factor determining the normal function and dysfunction of AF and IVD (Haglund et al., 2011). AF cells significantly respond to changes of hydrostatic pressure within AF. It was reported that AF cells could produce roughly 20% more proteoglycan under higher hydrostatic pressure (Le Maitre et al., 2008). On the other hand, the production of collagen I and aggrecan was found to reduce, while that of Tissue Inhibitor of Metalloproteinase-1 (TIMP-1) increased, which could affect remodeling of extracellular matrix (ECM) (Smith et al., 2004). This pressure sensing behavior appeared to be impaired in cells from degenerated AF as they responded less anabolically to physiologic intradiscal pressure (Wang et al., 2007; Wuertz et al., 2007). Shift from hydrostatic pressure to shear stress in the degenerated IVD was also reported to have distinct mechanobiological effects on AF cells. Similar to other load-bearing tissues like cartilage and bone, the increase in shear stress will stimulate the formation of fibrous tissues rich in collagen-I (Hsieh and Twomey, 2010). In addition, increased shear stress can also lead to the increasing of nitric oxide which could reduce the production of proteoglycan and increase apoptosis in the cells within IVD (Liu et al., 2001). Thus, the increased shear stresses in AF may accelerate IVD degeneration.

Biomechanical changes also induce inflammatory responses within AF and IVD (Purmessur et al., 2013). Indeed, in the altered biomechanical load model of degenerated IVD, significant increase was observed in the expression of inflammatory cytokines such as IL-1β, IL-6, TNF-α, and IL-8. These cytokines expressed by NP and AF cells remodeled the ECM from anabolism to catabolism, gradually leading to DDD (Le Maitre et al., 2005). IL-1 is up-regulated in degenerated human discs, inducing MMP-7, MMP-13, and ADAMTS, suggesting a deregulation of the normal IVD homoeostasis (Le Maitre et al., 2007). The biomechanically-induced inflammatory cytokines can also irritate the sensory nerves and cause pain response (Richardson et al., 2009). It is reported that TNF-α blockers had no effect on matrix degradation, but it might be contributing to discogenic pain in case where nerve ingrowth into IVD degenerative fissures occurs (Hess et al., 2011). By blocking p38 MAPK, it was possible to blunt the expression of factors associated with inflammation and to partially restore the synthesis of proteoglycans (Studer et al., 2008).

Overall, with the development of degeneration induced by altered biomechanical environment, there is a significant reduction in the expression of collagen-II and proteoglycans. Simultaneously, the expression of collagen-I also increases, indicating that matrix stress is changed and may in turn accelerate the progress of disc degeneration (Purmessur et al., 2013). This degenerative circle illustrates the progressive nature of DDD and serves as a backbone to improve scientific discussion and speed-up therapeutic advancement.

## Biological treatments of AF

Regarding to the multi-factorial nature of AF, three main therapeutic strategies have been practiced in the past, i.e., biomolecular-, nucleic acid-based therapies as well as intracelluar signaling pathways target therapy. All of these approaches have an long-term effects and the pro-anabolic approaches can trigger AF regeneration while lowering pain-related symptoms.

### Biomolecular therapies

Growth factors (GFs) are one of the indispensability effectors of the cell-cell communication. They are polypeptides that can bind with specific receptors on cells membranes and thereby influence cell behaviors such as ECM synthesis, proliferation and differentiation (Desai et al., 1999). The signaling by GFs can function in three different ways: paracrine (a signal produced by a cell to induce changes in nearby cells), autocrine (a hormone or chemical messenger secreted by a cell to induce self changes) and endocrine (a hormone secreted into the blood circulation directly by a specific organ) (Wang et al., 2003). Delivery of GFs to mobilize endogenous cells probably becomes an effective solution than allogeneic or autologous cell transplantation to reestablish homeostasis in an early degenerated AF. The studies summarized in Table 1 show promising results after injection of various GFs, such as transforming growth factor-β1 (TGF-β1), TGF-β3, bone morphogenetic protein-2 (BMP-2), BMP-7, epidermal growth factor (EGF), basic fibroblast growth factor (bFGF), insulin-like growth factor-I (IGF-I), and platelet-derived growth factor (PDGF) (Tolonen et al., 2006).

**Table 1:** Growth factors and gene therapy approaches for AF regeneration.

**Growth factor/gene**	**Approach**	**Model**	**Finding**	**References**
BMP-2 and TGF-β1	Exposure of AF cells to combination of BMP-2 and TGF-β	*In vitro*	Aggrecan content ↑	Cho et al., 2013
TGF-β1, IGF-1 and BMP-2	IVD cells were transducted with GFs by using an adenoviral vector	*In vitro*	Proteoglycan content↑	Moon et al., 2008
SOX-9	Intradual injection of SOX-9 by using adenoviral vector	*In vivo*	Collagen and proteoglycans content ↑	Paul et al., 2003
SOX-9 and OP-1	Double gene transfection by using an adeo-associated virus	*In vivo*	Col II and proteoglycans content ↑	Ren et al., 2013
OP-1	IVD cells were exposed to Chondroitinase and OP-1	*In vitro*	proteoglycans content ↑	Chubinskaya et al., 2007
BMPs and SOX-9	Tranduced NP cells of BMPs and SOX-9 by using adenovirus	*In vivo*	Collagen and proteoglycans content ↑	Zhang et al., 2007
BMP-2	AF and NP cells cultured in the presence of BMP-2	*In vitro*	Collagen and aggrecan contend↑	Kim et al., 2003
BMP-3	AF cells were exposed to BMP-3	*In vitro*	Collagen and aggrecan contend↑	Li et al., 2008
GDF-5	Human adipose stem cells were stimulated by GDF-5	*In vitro*	Col II and aggrecan content↑; MMP-3 content ↓	Hoogendoorn et al., 2008
VEGF	AF cells were cultured in the presence of VEGF	*In vitro*	CX40, CX43 content↑	Dezawa, 2008
bFGF	IVD cells were exposed to bFGF	*In vitro*	Perlecan content↑	Yoshimura et al., 2007

Recently, the crucial role of GFs in AF physiology was more proven, and the application of GFs as therapeutic approaches to retard, treat or reverse the process of AF degeneration could be of great potential. Studies on the effects of GFs on cell behavior showed that intradiscal injections of GFs increased ECM production. As reported, IGF-1, and TGF-β1 could stimulate the synthesis of sulphated GAGs, collagen I, collagen II, and reduce activation of matrix metalloproteinase-2 (MMP-2) and MMP-13 in AF cells, as a result of facilitating cells to present a fibrocartilagious phenotype (Moon et al., 2008). GDF-5 is a major factor involved in early IVD development, it favors cell proliferation, and stimulates the synthesis of collagen-II and proteoglycan *in vitro*. Intradiscal injection of GDF-5 in rabbits and rats was shown to increase disc height. In addition, clinical trials were conduct to evaluate the safety and efficacy of intradiscal injection of GDF-5 in patients with DDD (NCT01124006, NCT01158925 and NCT01182337 on clinicaltrials.gov), however, no peer-reviewed publication is currently available. While a modest improvement in clinical parameters was observed, some researchers suggested that direct intradiscal injection may not be the most suitable route (Blanquer et al., 2015).

In fact, it is likely that GFs act synergistically in promoting anabolic activity of cells. Therefore, effective clinically use of GFs may potentially be obtained by cocktails of them. To design an *in vitro* model of AF degeneration, Cho et al. applied AF cells that were cultured in monolayer with the stimulation of IL-1β and TNF-α. Researchers analyzed the synergistic effects of TGF-β1 and BMP-2 and compared with administration of either GF alone. Results showed that the combination of the GFs effectively increased the anabolism and decreased the catabolism of AF cells, indicating that the use of cocktails of GFs could provide better outcomes than either alone (Cho et al., 2013).

Despite the therapeutic significance of GFs, several limitations remain to be overcome. Due to the different cell density and populations within AF, it is much more difficult for AF regeneration compared with other tissues. Furthermore, the level of degeneration significantly affected the success of GF therapy as the degenerative AF may lead to down-regulation of ECM synthesis and cell apoptosis (Elmasry et al., 2016). In addition, the half-life of GFs is generally short. Therefore, multiple injections of GFs may be required in order to obtain long-term effects, which may lead to a high risk of unpredictable side effects. Undesirable side effects such as ectopic bone formation after direct injection of BMP may accelerate the degeneration progress (Michalek et al., 2010). Meanwhile, as the internal pressure of disc could bring about leakages in the adjacent tissues, injection of GFs in a liquid state is not considered to be an ideal form of delivery because of its undesirable side effects. Accordingly, gene therapy, and sustained delivery systems are being developed to improve the local delivery of GFs in order to enhance the efficacy of the outcomes.

### Gene and interfering RNA therapies

Delivery of genes into a patient's AF cells using short fragment of DNA can potentially manipulate the synthesis and secretion of encoded proteins to enhance AF homeostasis and possibly suppress degenerative disease (Chadderdon et al., 2004). In terms of the apoptosis of cells in degenerated AF, direct gene therapy can be appropriate for early degenerative stage, while it appears that indirect method is of more interest for more severe cases. One obvious advantage of genetic modification method is the prolonged lasting effect compared with repeated injection of therapeutic GFs, which maintain a desired concentration of GFs for an extended period. However, owing to the very limited cell capacity to uptake genetic material, gene therapy needs vectors to deliver genetic material into the cells (Shimer et al., 2004). Two main methods of gene delivery can be envisioned: (i) *In vivo* gene therapy, the vectors conveying the modified genes can be introduced in the body directly or (ii) *ex vivo* gene therapy, target cells can be harvested, genetically modified *in vitro*, and re-implanted. Two kinds of carriers are mainly used for gene, non-viral and viral vectors delivery. For gene therapy of AF, most studies used adenovirus and adeno-associated virus (AAV) as the vectors. It was found that the use of adenoviral vectors coding for TIMP-1 and BMP-2 on human degenerated disc cells increased proteoglycan synthesis (Wallach et al., 2003b). In addition, the efficacy of AAV-mediated co-transfection of Sox-9 and OP-1 genes were tested in a rabbit disc degeneration model, and the MRI signal, disc height, gene expression, and matrix molecules were found to increase dramatically (Ren et al., 2013). A mass of viral vector delivery systems were evaluated. However, the most prominent problem of viral vehicles is the potential infection and immunological rejection (Wallach et al., 2003a). Non-viral gene therapy involves the use of cationic compounds or naked DNA including liposomes and “gene guns.” There are limited reports on non-virus mediated gene transfer applications in the IVD. Nishida et al. achieved sustained transgene expression in IVD cells *in vivo* through a microbubble-enhanced ultrasound gene therapy (Nishida et al., 2006). Although the therapy might be the preferred for lower infection possibility, non-viral gene delivery systems are generally of insufficient transfection efficiency. More recently, many investigators developed new more efficient protocols to overcome the limitations of non-viral gene delivery systems (Dash et al., 2011; Mahor et al., 2011). It is promising that non-viral gene delivery systems will draw increasing attention in future therapies for disc diseases.

Gene therapy based on siRNAs is a recently-developed parallel strategy to classic gene therapies (Woods et al., 2011). SiRNAs can control the gene expression by binding to target mRNA and inhibiting RNA degradation or translation. Preliminary studies that investigated the use of siRNA for transfecting disc cells have shown some promising results (Clouet et al., 2018). In an *in vitro* inflammation model in which AF and NP cells were activated by LPS treatment to mimic their inflammatory phenotypes in DDD, the cells were transfected with IL-1β siRNAs by a polymeric system. Such treatment significantly knocked down the expression of IL-1β. In addition, the inhibitory effect was more pronounced in AF cells than in NP cells (Newland et al., 2013).

To the best of our knowledge, there was no clinical trial based on gene or siRNA therapies for AF regeneration. Despite encouraging results, these approaches need further improvement considering harsh environment where these biological molecules were injected. Therefore, using biomaterials to protect these molecules and nucleic acids can be an effective strategy. The biologics loaded by biomaterials can be delivered in a spatiotemporally controlled manner so as to promote long-term regeneration of AF.

### Intracellular signaling pathways as therapeutic targets

In the recent years, numerous intracellular signaling pathways have been focused on AF degeneration. Intracellular signaling controls most of cellular activities to maintain homeostasis in harsh environment. Studies showed that nuclear factor kappa-B (NF-κB) pathway, mitogen-activated protein kinase (MAPK), and Wnt pathway play crucial roles in adaptation of AF cells, pain mediators and IVD degeneration. Active MAPK and NF-κB signaling can lead to production of matrix degrading enzymes such as MMPs and aggrecanases. A study investigated the roles of MAPK and NF-κB pathways in regulating pro-inflammatory mediators such as TNF-α, IL-1β or IL-6 in several musculoskeletal disorders (Klawitter et al., 2012). MAPK pathway was also identified as a potential therapeutic target for control of anti-catabolic and anti-inflammatory situations in IVD. Suppression of MAPK pathway could also regulate integrin expression, matrix production, osmosis, and cell survival of IVD cells (Yang et al., 2016). Inhibition of p38 MAPK successfully suppressed secretion of pro-inflammatory cytokines while restoring proteoglycan synthesis in AF cells. Likewise, NF-κB activity has also been considered a potential therapeutic target to treat IVD hernitation and associated pain. Increased NF-κB activity was correlated with reduced proteoglycan content in IVD. Blocking NF-κB signaling pathway by inhibitor could improve the accumulation of proteoglycan and was able to counteract the apoptosis of disc cells and matrix deposition in animal models (Morwood and Nicholson, 2006).

Wnt pathway is also associated with the progression of DDD. Wnt signaling follows either the classical Wnt/β-catenin-dependent pathway or the non-classical β-catenin-independent pathway (Kondo et al., 2011). In a study examining the Wnt signaling in IVD, treatment of IVD cells with LiCl, an activator of Wnt, led to an up-regulation of β-catenin, and induced a decrease in cell proliferation (Hiyama et al., 2010). Another study investigated the DDD in β-catenin conditional mice. It seemed that the expression levels of catabolic genes such as aggrecanase and MMP-13 were up-regulated in disc cells of β-catenin knockout mice (Hiyama et al., 2011). These findings suggest that Wnt/β-catenin-dependent pathway might play a key role to maintain the function of IVD.

Recently, the Notch signaling pathway, a hypoxia sensitive transducer, was reported to be closely related to proliferation of progenitor cells in harsh environments of IVD. The proportion of Notch signaling can be activated by hypoxia in IVD cells. Inhibition of Notch signaling blocked hypoxic induction of the activity of the Notch-responsive luciferase reporters and reduced AF cell proliferation. In addition, Notch signaling may play an important role in degenerative processes mediated by pro-inflammatory cytokine (Wang et al., 2013). In degenerate discs, the expression of Notch-2 and IL-1β was significantly increased compared with non-degenerate discs.

More recently, Piezo protein was proved to be a vital mechanical regulator, which had a preponderant influence on maintaining AF tissue homeostasis. Researchers demonstrated that Piezo promoted differentiation of secretory enteroendocrine cells by inhibition of Notch signaling (Sugimoto et al., 2017). Thus, Piezo signaling might become another important factor on proliferation, differentiation, and apoptosis of AF.

## Mechano-regulation of stem cells toward AF regeneration

Cells are the basic components of biological tissue, which largely determines the properties and functions. To encounter the decreasing cells during AF degeneration, repopulating degenerated AF with healthy cells aims to restore tissue homeostasis by transplanting cells able to secrete functional ECM. Various cells were considered and tested to achieve these clinically objectives. NP and AF cells harvested from surgically removed IVD were first used in human clinical trials with promising results (Gruber et al., 2004). However, the low number of harvested cell limited their further use. Stem cell engineering provides an alternative way to address this issue. With appropriate stimulation, stem cells can be directed to differentiate toward various specific cell phenotypes. Bone marrow derived mesenchymal stem cells (MSCs) and adipose derived stem cells (ASCs) in their undifferentiated state are currently the most widely used cell types (Yañez et al., 2006; Elsaadany et al., 2017). MSCs are considered as immune-evasive and hypo-immunogenic that could be transplanted across major histocompatibility barriers. ASCs are abundant in fat tissue, with advantages of easy accessibility, and more potent immune-modulatory effects in comparison with MSC. These cells were also reported to secrete GAGs and collagen-II when implanted in mice (Hoogendoorn et al., 2008). In animal models such as rabbit, rat and mouse, MSCs injected in the IVD successfully inhibited disc degeneration by reducing the expression of catabolic genes and promoting the synthesis of anabolic genes, resulting in the maintenance of the AF integrity. Recently, pluripotent stem cells and induced pluripotent stem (iPS) cells were also considered for AF regeneration for their theoretical potential to differentiate into AF cells (Kraus and Lufkin, 2017). In the past few years, our group isolated a population of AF-derived colony-forming cells from rabbit IVD. These cells were self-renewable and could be readily induced to differentiate into different cell types including osteocytes, chondrocytes, and adipocytes, respectively. Such AF-derived stem cells (AFSCs) could potentially be a valuable source for repair or regeneration of AF tissue (Liu et al., 2014). In addition, we found that transforming growth factor-β3-mediated BMSC had strong tendency to differentiate into various types of AF cells and presented gene expression profiles similar to AFSCs (Guo et al., 2015).

Mechanical stimulus is one of the most effective stimuli in regulating the differentiation of stem cells (Doroski et al., 2010; Li and Wang, 2010). Applied forces, including compression, tension, hydrostatic pressure, and stress, could remarkably affect the maintenance and lineage specification of MSCs (Discher et al., 2009). It is believed that a diversity of mechanical signals within *in vivo* niches work together to regulate the fate of stem cells. For example, tensile forces induce osteogenic differentiation while compression forces can lead to chondrogenic differentiation. External mechanical stimuli also influence the growth of cells and formation of ECM. Elsaadany et al. applied varying magnitudes of equiaxial strain at different frequencies to investigate the differentiation of ASCs (Elsaadany et al., 2017). They found that 6% strain and 1 Hz was the optimal loading modality to induce differentiation into AF-like cells and formation of AF-like matrix. Furthermore, the equiaxial load also induced region-specific differentiation of ASCs in the inner and outer regions on their scaffolds. In our recent work, we applied cyclic tensile strain (CTS) with different magnitudes (2, 5, and 12%) during AFSCs culture. The expression of anabolic genes (collagen-I, collagen-II, and aggrecan) in AFSCs was found to increase with increasing applied CTS from low (2%) to moderate (5%) at a frequency of 0.5 Hz for 4 h. In contrast, the anabolic genes expression decreased at high magnitude of CTS (12%). In rabbit injected with stimulated AFSCs (CTS = 5%) in AF, IVD degeneration was found to be significantly slowed down, and disc height and MRI signal intensity were dramatically improved (unpublished data).

Recent advances also highlighted the critical role of internal forces due to cell–matrix interaction (Engler et al., 2006). MSCs were found to be regulated by internal microscopic forces that occurred on contracting cells pulled by each other or by surrounding ECM. These internal forces can be regulated by adjusting the properties of scaffold materials, such as elasticity, microstructure, and substrate patterning (Zhu et al., 2016). The seeded cells can generate contractile forces to sense the properties of substrates and thereby perceive mechanical information that directs the fate of MSC such as proliferation, migration, and differentiation. More importantly, a moderate mechanical regulation is found to be required to stimulate the anabolic expression, which can potentially be utilized for repair and regeneration of AF.

Despite the global promise of cell therapies, the stringer conditions of DDD (low nutrient level, high osmolarity, low oxygen tension, and high intradiscal pressure) hampered the application of therapy by injection of stem cells. To overcome it, engineered scaffolds may be used to create a biomimetic environment that mimics the native structure and mechanical properties of AF. These scaffolds can additionally limit the risk of off-target effects related to cell effusion by promoting cell retention at the injection site.

## Scaffolds for AF tissue engineering

Tissue engineering, by combining material science and biological engineering, is a promising way to restore the normal functions of tissues and tissue regeneration (Laschke et al., 2009). Scaffolds are one of the most important elements in AF TE by providing appropriate mechanical properties, adequate space, and biochemical cues for seeded cells to grow, differentiate, and produce ECM to regenerate AF tissue (Yeganegi et al., 2010). Ideally, the scaffold should mimic the native AF tissue that should be strong enough to sustain the complex mechanical loads after implantation. Meanwhile, the scaffolds should be fully biodegradable and allow the maintenance of cell phenotype. In addition, they should mimic the microstructure and anisotropic biochemical properties of native AF tissue, allowing an appropriate delivery of bioactive agents, and be suitable to fixation.

**Figure 2:**
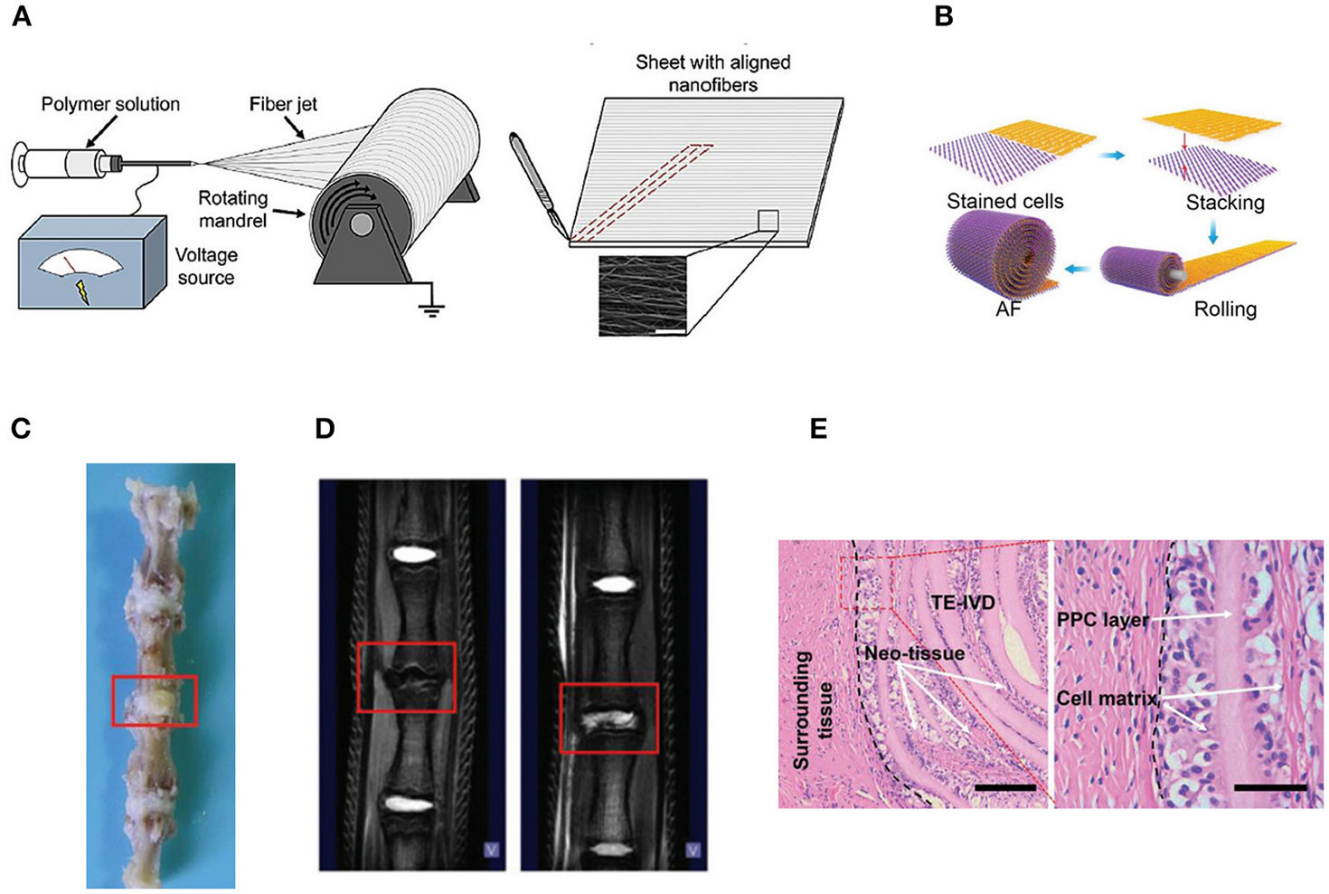
**(A)** Electrospinning of polymer solutions is used to create aligned fibrous sheets that are then wound circumferentially to yield an aligned AF to encase the NP (Reprinted with permission from Bowles and Setton, 2017). **(B)** The schematic shows the process of building artificial AF by constructing multilamellar PCL/ PLGA/Collagen I (PPC). **(C)** The TE IVD implant integrates with the native vertebrae. **(D)** The MRI images of the control (Left) and PPC IVD (Right) after implantation. **(E)** H&E staining shows the interface between the PPC IVD and the surrounding tissues (Reprinted with permission from Yang et al., 2017).

Various kinds of scaffolds have been designed for AF engineering. The scaffolds can be made from natural materials or synthetic materials. Nature materials include collagen, hyaluronic acid (HA), chitosan, alginate, silk fibroin, and chondroitin sulfate (CS). Some researchers also paid attention to natural biologic materials such as decellularized matrix from bone, AF, or IVD, fibrinogen isolated from bovine plasma and bone matrix gelatin (BMG) (Chan et al., 2013; Pereira et al., 2018). Obtained by bacterial fermentation or extracted from natural sources, the natural scaffolds are endowed with advantages such as low toxicity, similarity to native tissue, and easy large-scale production. Synthetic polymers are obtained from reproducible industrial products and their mechanical and physicochemical properties can be finely adjusted. The most commonly synthetic materials used for AF TE include poly(trimethylene carbonate) (PTMC), poly(lactide-co-glycolide) (PLGA), poly(ε-caprolactone) (PCL), poly(D, L-lactide) (PDLLA), poly(L-lactide) (PLLA), polyurethane, and HA-poly(ethylene glycol) (PEG) (Nerurkar et al., 2011; Vadalà et al., 2012) (Table 2). These scaffolds can be fabricated and processed using various techniques depending on the desired structure characteristics (aligned, angle-ply, hierarchical, bilayer, biphasic, etc) and mechanical properties of the final engineered tissue. Among the techniques, electrospinning is preferred for AF TE by researchers for its ability to produce micro- and nanofibers which largely recapitulate the structural characters of native AF tissue (Figure 2). In addition, diverse bioactive substances, such as proteins, antibiotics, can be encapsulated in the scaffolds to realized controlled delivery of bioactive molecules to enhance AF regeneration.

**Table 2:** Composite strategies for AF TE. [Reproduced with permission from (Li et al., 2017)].

**Scaffold**	**Cell source**	**Environment**	**Bioactive agents**	**ECM expression**	**Mechanical property**	**Representative figure**	**References**
Electrospun PCL, aligned	Calf BMSCs	*In vitro*	—	sGAG and collagen content ↑	Uniaxial tensile moduli of MSC-seeded parallel and opposing bilayers increased	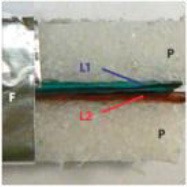	Nerurkar et al., 2009
Electrospun Polyurethane, aligned	Bovine AFCs	*In vitro*	—	—	Both the initial modulus and tensile strength of aligned scaffolds were higher than random fiber scaffolds	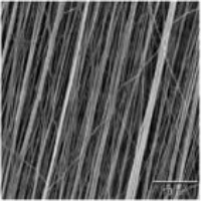	Yeganegi et al., 2010
Electrospun PCL, aligned	Porcine AFCs	*In vitro*	—	Proteoglycan, collagen, and DNA content ↑	—	—	Nerurkar et al., 2011
Electrospun PCL, aligned	Bovine BMSCs	*In vitro*	—	Collagen and GAG content↑	GAG was important for compressive properties while Collagen dominated the tensile response	—	Koepsell et al., 2011
Electrospun PLLA, meshwork	Bovine AFCs	*In vitro*	TGF-β1	Collagen and GAG content↑	—	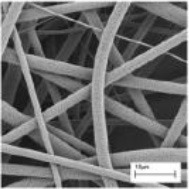	Vadalà et al., 2012
Electrospun PCL, meshwork	porcine AFCs	*In vitro*	berberine	sGAG and collagen content↑	—	—	Driscoll et al., 2013
Electrospun PGA and PVDF, meshwork	—	*In vivo*	—	—	—	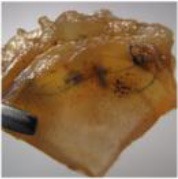	Hegewald et al., 2013
Electrospun PLLA, Angle-ply, hierarchical	—	*In vivo*	—	—	—	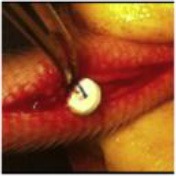	Martin et al., 2014
Electrospun Polyurethane, aligned	Bovine AFCs	*In vivo*	—	Col-I and TGF-β1 content↑	Scaffold elastic modulus increased on day 7	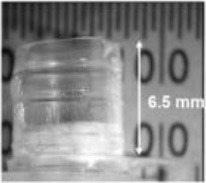	Turner et al., 2014
Decellularized rabbit bone matrix, 3D	Rabbit AFCs	*In vivo*	—	Proteoglycan, collagen, and DNA content ↑	—	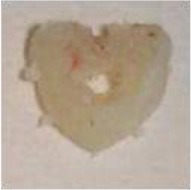	Pan et al., 2012
Decellularized, Porcine AF tissue, 3D	Porcine AFCs	*In vitro*	—	—	Elastic moduli was almost equal control group	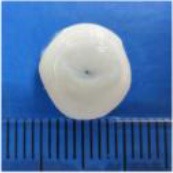	Chan et al., 2013
Lyophilized SF, biphasic scaffold	Porcine AFCs	*In vivo*	—	Collagen and GAG and DNA content ↑	—	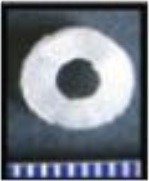	Park et al., 2012a
Lyophilized SF, 3D	Porcine AFCs	*In vivo*	—	Collagen and GAG and DNA content ↑	Elastic modulus of porous and lamellar scaffolds reached similar values after 2 weeks	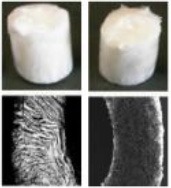	Park et al., 2012b
Salt-leaching and penetrating PLGA and HA, 3D	Rabbit AFCs	*In vitro*	—	GAGs, and collagen content↑	—	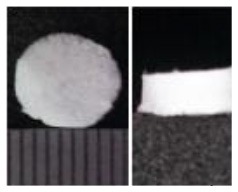	Wu et al., 2010
PLGA nanoparticles dispersed in a dextran/gelatin hydrogel, 3D	Mouse MSCs	*In vitro*	TGF-β3	Col II and aggrecan content ↑	—	—	Gan et al., 2016
Rod-shaped colloidal silica nanofibers associated with hydrogels	Human adipos stromal cell	*In vitro*	TGF-β1 and GDF-5	—	—	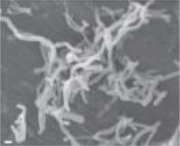	Henry et al., 2017

### Microstructure and mechanical characteristics of scaffolds

The microstructure of scaffolds significantly affects the function and morphology of cells cultured on it. By replicating the key length scales and structural features of native AF tissues, aligned fibrous scaffolds are currently preferred for AF regeneration, which are expected to control cell morphology and differentiation, as well as direct the ordered deposition of new ECM. Many studies have shown that fibers orientation can affect the final mechanical properties and the matrix formation of engineered AF constructs. Our group recently found that aligned fibrous scaffolds could provide a favorable microenvironment for AFSCs to differentiate into cell phenotypes similar to AF cells in different regions (Liu et al., 2015). Nerurkar et al. fabricated aligned nanofibrous scaffolds with nonlinear distribution of modulus mimicking that of native AF. They found that AF cells grew along the fibers within scaffolds and formed ECM with considerable alignment (Nerurkar et al., 2009). In a study using silk fibroin (SF) based scaffolds with fibers alignment resembling the fibrous orientation of AF tissue, the scaffolds guided the alignment of human chondrocytes and, in turn, the alignment of the deposited ECM (Bhattacharjee et al., 2012). By offering the combined effects of cell/matrix alignment and chondrogenic re-differentiation support, these aligned SF scaffolds can potentially serve as a suitable substrate for AF regeneration. In all, the topographical and structural features of scaffolds can regulate cell behaviors such as division, matrix synthesis, and apoptosis, and can even direct stem cells to differentiate toward specific lineages.

In addition to composition and structure, the mechanical property of scaffold is also an important designing factor for AF TE (Yeganegi et al., 2010; Nerurkar et al., 2011). An ideal AF substitute should recapitulate the mechanical properties and distribution of native AF tissue. The mechanical properties such as stiffness can significantly affect the cell behaviors such as adhesion, proliferation, differentiation, and migration, and thereby play important roles in AF repair and regeneration. In particular, the stiffness of substrate was found to affect the differentiation of stem cells and direct their lineage specification. Similarly, rat AF cells were reported to be sensitive to substrate stiffness which could regulate their morphology, growth, apoptosis, and ECM metabolism (Zhang et al., 2011). In our recent studies, we fabricated a series of nanofibrous polyurethane scaffolds with elastic modulus close to that of native AF tissue and studied the behaviors of AFSCs and BMSCs seeded on the scaffolds. Depending on the elasticity of scaffold materials, AFSCs showed strong tendency to differentiate into different AF-like cells. On scaffolds with low modulus, the expression of collagen-I in both AFSCs and tBMSCs was relatively low, while the expression collagen-II, and aggrecan was relatively high. The opposite trend was observed on scaffolds with high modulus (Guo et al., 2015; Zhu et al., 2016). Since the inner region of AF mainly consists of collagen-II and PGs and outer regions mainly contains collagen I, it was likely that both AFSCs and tBMSCs tend to differentiate into cells in inner region of AF on the soft scaffolds, whereas they preferred to differentiate into cells in outer region of AF on the stiff scaffolds. Wan et al. fabricated a biphasic elastic scaffold to elastically and structurally simulate the AF. The inner phase of the scaffold was an elastic material based on poly (polycarolactone triol malate) (PPCLM), while the outer part of the scaffold was demineralized bone matrix gelation (BMG). The biphasic scaffold had an enhanced compressive strength and tensile stress than uniphasic scaffold, making it a promising candidate for AF repair (Wan et al., 2008). In all, the mechanical properties of scaffolds remarkably affects the biochemical and biomechanical properties of cultured AFSCs and the ECM they produce. These findings provide new insights toward developing engineered AF with biological characteristics and mechanical functions approximating the native AF tissues.

### Delivery of bioactive agents using tissue engineering scaffolds

Scaffolds have been widely used for AF TE. However, limited studies reported the use of scaffolds in combination with the delivery of bioactive agents for AF regeneration. Vadala et al. reported a bioactive electrospun PLLA scaffold containing TGF-β1, which promoted collagen and glycosaminoglycan production and cell proliferation (Vadalà et al., 2012). The half-life activity of TGF-β1 is generally no longer than 2–3 min, and thereby repeated injections are needed which can lead to unwanted side effects like toxicity. The release profile of TGF-β1 from the PLLA electrospun matrix showed a sustained delivery, avoiding the toxic side effects. To enhance AF regeneration, Guillaume et al. developed a biomimetic poly (trimethylene carbonate) (PTMC) scaffold with controlled pore characteristics and combined it with sustained delivery of active compounds as TGF-beta 3 and FGF-2 (Guillaume et al., 2014). Bao et al. reported the incorporation of anti-microbial agents such as berberine in electrospun PCL scaffolds with a sustained strong anti-fungal and anti-bacterial activity for 33 days (Bao et al., 2013). Overall, combining scaffolds with drug delivery capacity is attracting increasing attention for AF engineering.

## Concluding remarks

Effective AF repair is a clinical demand for treating DDD. With the massive findings recently, the physiopathology of AF degeneration has been gradually unveiled. Various biotherapies have been proposed, including molecular therapies, nucleic acid-based therapies and mechano-regulated cell based therapies. These therapies, aiming at supplementing biologics including GFs, genes and cells in AF, have shown promising results *in vitro* and *in vivo*. Nevertheless, their clinical uses still remain a major concern due to the short-term efficacy and insufficient stability of them. These limitations may be, at least partially, overcome by biomaterials-based TE using a combination of cells and biomolecules to restore AF anabolism. In addition, advanced drug delivery approaches that suit specifically for AF TE will also be a promising strategy for clinical translation.

In recent years, interdisciplinary strategies addressing biologic, biomechanical, and biomaterial needs for AF regeneration were initiated. Contributions from all these areas are important to construct a better biological implantation, which is expected to replicate the properties of AF and help maintain the mechanical properties of the IVD after implantation. AF regeneration relies on a comprehensive consideration of biological, biomechanical, and biomaterial cues. Future development should pay more attention to making full use of mechanical stimulation or biomaterials-mediated delivery of biomechanics to effectively achieve AF regeneration. Continued exploration of intracellular signaling pathways of biomechanical and biomaterials-mediated mechano-transduction should provide novel approach to restoring the homeostatic mechanical properties of AF and ultimately to achieving effective treatments for DDD.

## Author contributions

BL designed the topic and main structure and wrote the manuscript. GC, CS, HW, WZ, and HY wrote the manuscript.

### Conflict of interest statement

The authors declare that the research was conducted in the absence of any commercial or financial relationships that could be construed as a potential conflict of interest.
